# Supplementary figures and images for: Characterization of factors that underlie transcriptional silencing in C. elegans oocytes
Source: PLoS Genet. 2023 Jul 21;19(7):e1010831. doi: 10.1371/journal.pgen.1010831 (PMC10395837; doi:10.1371/journal.pgen.1010831)

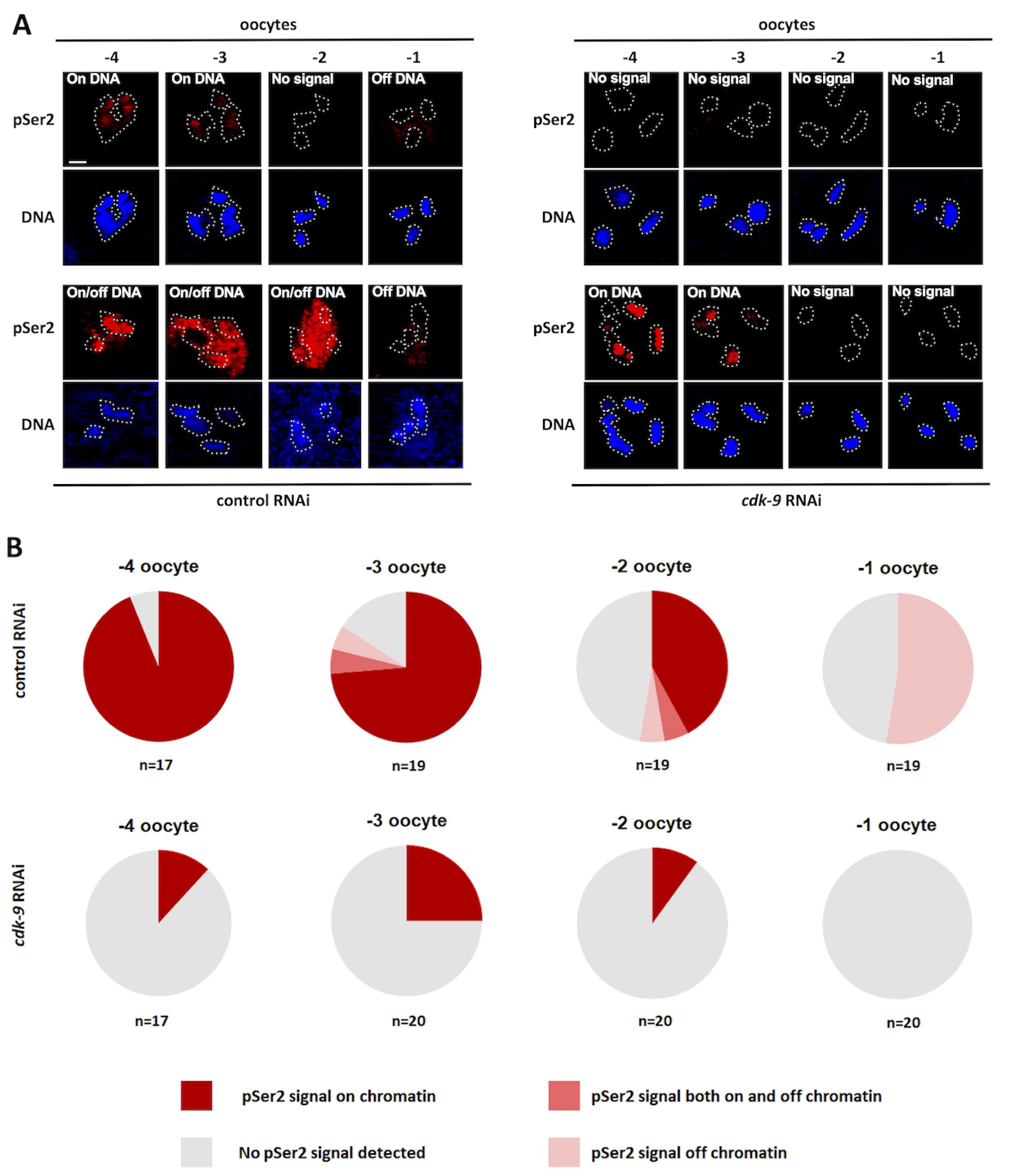

Supplement: S1 Fig — A. The different patterns of RNAPIIpSer2 (red) signal on and/or off DNA (blue) observed in N2s treated either control or cdk-9 RNAi. Depletion of CDK-9 results in the loss of RNAPIIpSer2 signal in proximal oocytes regardless of RNAPIIpSer2 signal localization. Scale bar represents a length of 2 μm. B. Visualization of data presented in (A). N2s treated with cdk-9 RNAi showed reduced RNAPIIpSer2 signal in all forms. The number of samples analyzed over 2 independent replicates is presented below the charts for each oocyte position. (TIFF) [file pgen.1010831.s001.tiff]

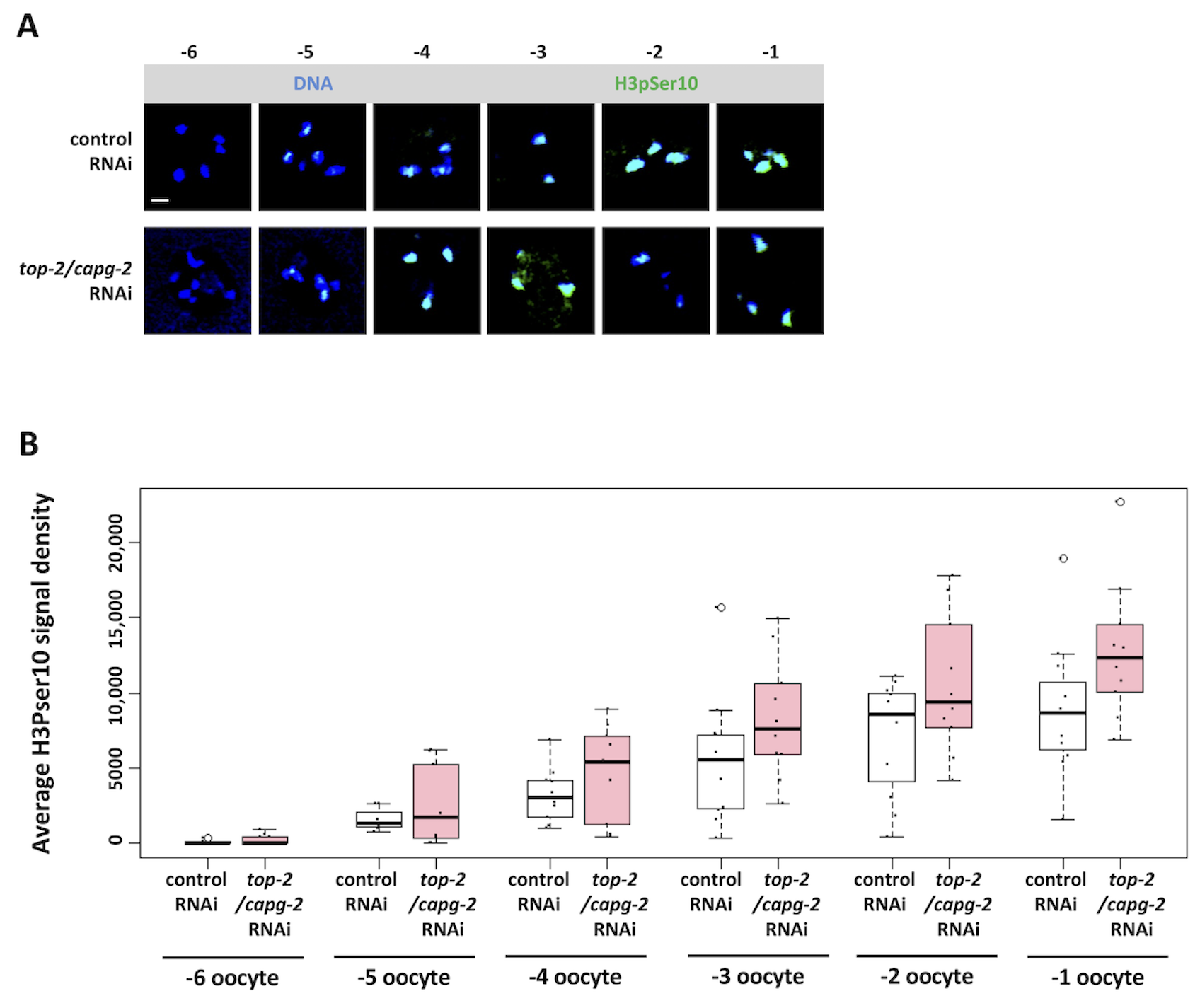

Supplement: S2 Fig — A. N2 animals were treated with control or top-2/capg-2 double RNAi. Gonads from young adults were dissected, fixed, and stained for DNA (blue) and H3pSer10 (green). Exposure to top-2/capg-2 RNAi does not affect the timing of H3pSer10. Scale bar represents a length of 2 μm. B. Quantification of data presented in (A). 11 control RNAi and 10 top-2/capg-2 RNAi samples were analyzed for each RNAi treatment. There was no significant difference in H3pSer10 signal after TOP-2 and CAPG-2 co-depletion. Signal density was not normalized. (TIFF) [file pgen.1010831.s002.tiff]

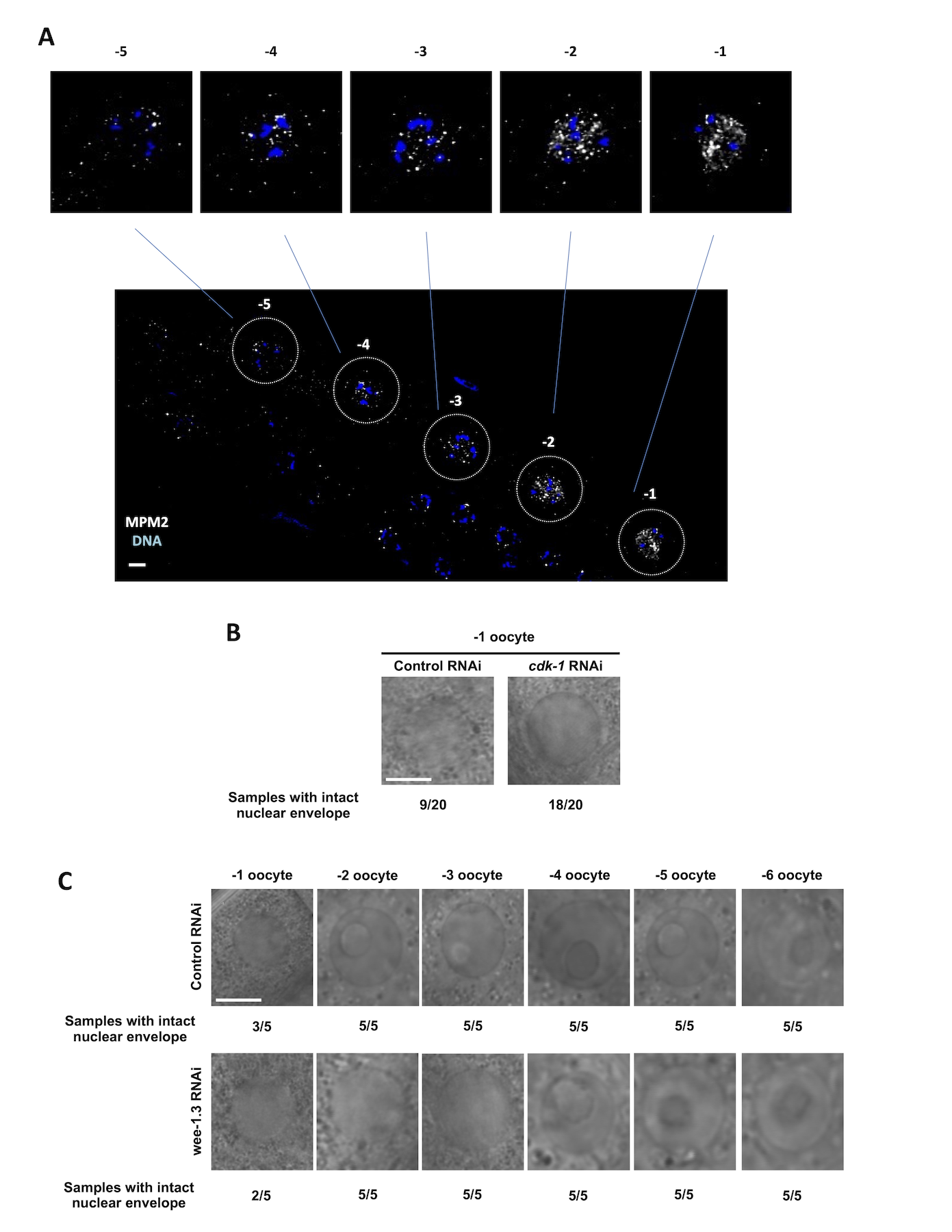

Supplement: S3 Fig — A. N2 gonads were dissected, fixed, and stained for DNA (blue) and MPM-2 (white). An increase in MPM-2 signal is observed at the proximal oocyte positions in comparison to more distal oocytes. Scale bar represents a length of 2 μm. B. N2 samples were treated with either control or cdk-1 RNAi. Nuclear membrane of -1 oocytes were evaluated using live phase-contrast microscopy. 20 samples were analyzed over two replicates and quantifications are presented below each representative image. cdk-1 RNAi resulted in a higher number of samples with intact nuclear envelope when compared to control RNAi treatment. C. N2 samples were treated with either control or wee-1.3 RNAi. Nuclear envelope integrity was evaluated like in (B) for oocytes in -1 to -6 positions. 5 gonads were analyzed for each treatment and the quantifications are presented below each representative image. Treatment with wee-1.3 RNAi did not alter the progression of NEB in proximal oocytes. (TIFF) [file pgen.1010831.s003.tiff]

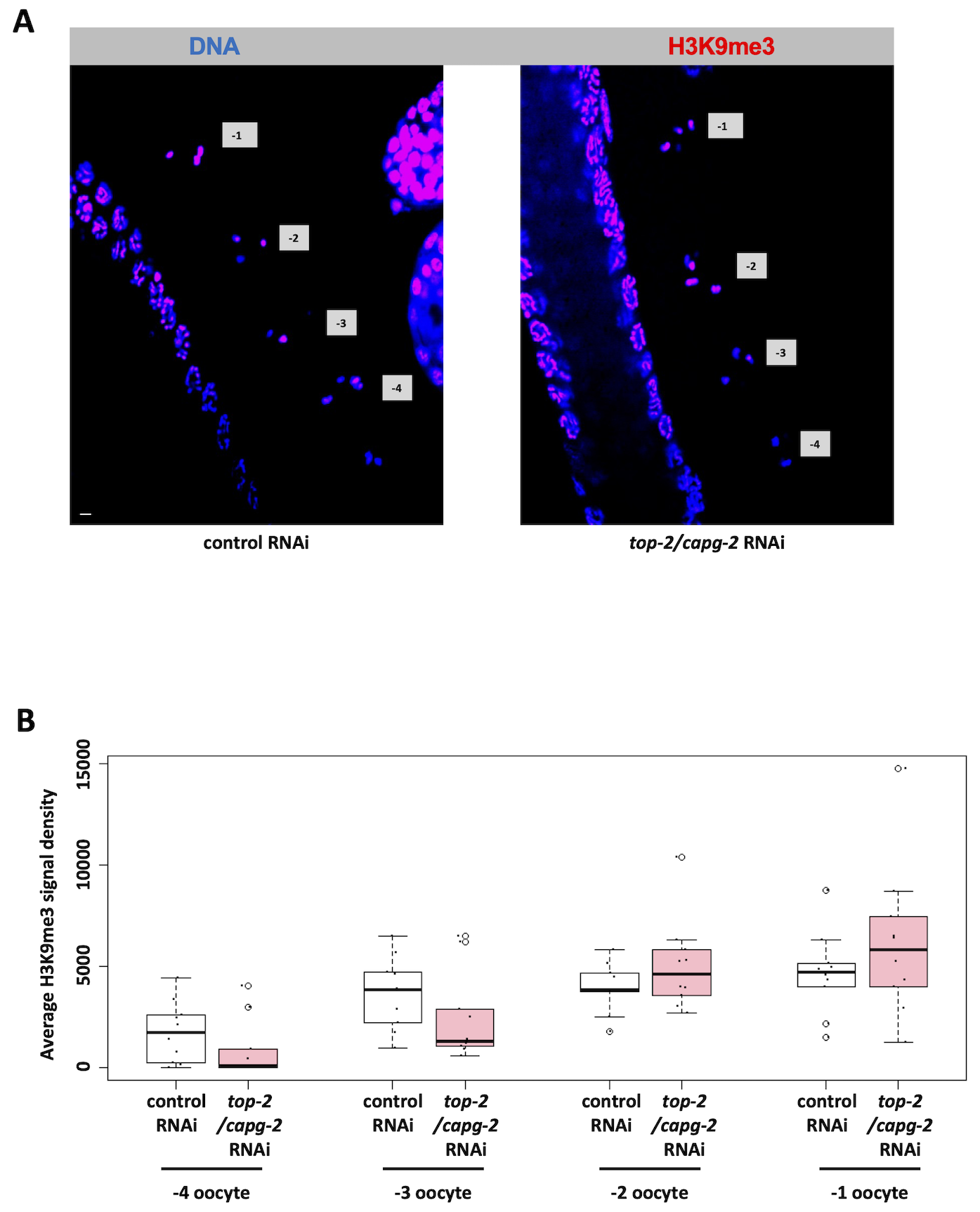

Supplement: S4 Fig — A. Gonads from N2 adults treated with either control or top-2/capg-2 RNAi were dissected, fixed, and stained for DNA (blue) and H3K9me3 (red). Treatment with top-2/capg-2 RNAi did not alter the deposition of H3K9me3 in proximal gonads. Scale bar represents a length of 2 μm. B. Quantification of signal density for the data presented in (A). 10 samples were analyzed for each treatment. H3K9me3 signal remained the same when top-2/capg-2 RNAi treated samples were compared with those treated with control RNAi. (TIFF) [file pgen.1010831.s004.tiff]

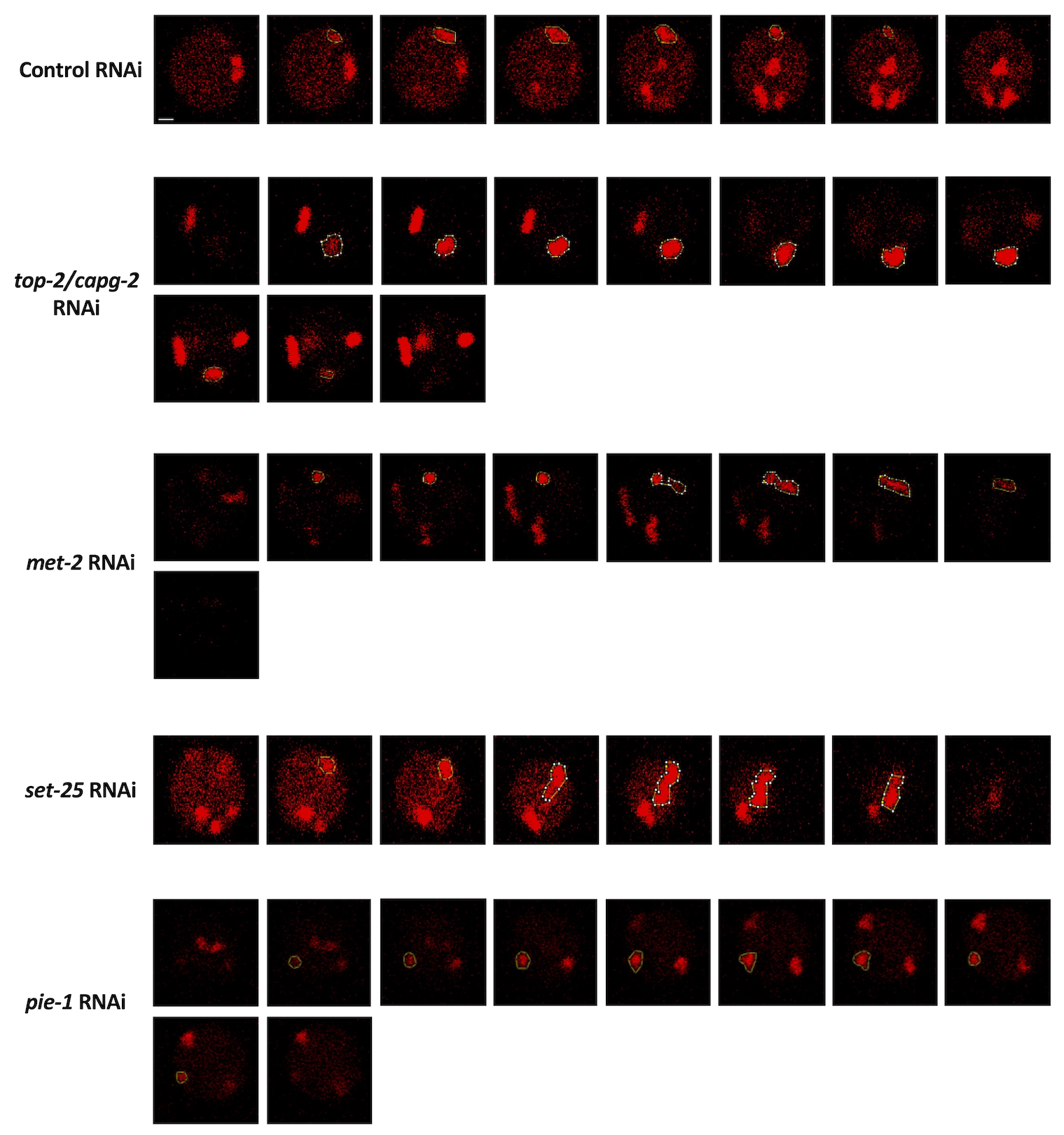

Supplement: S5 Fig — Z-stacks were taken from living oocytes at the -2 position in animals treated with control, top-2/capg-2, met-2, set-25, and pie-1 RNAi. Shown here are representative images of a bivalent volume measurement from each RNAi treatment. See Methods for details on bivalent volume measurement. Scale bar represents a length of 2 μm. (TIFF) [file pgen.1010831.s005.tiff]
